# Comparative transcriptome analysis reveals that PCK1 is a potential gene affecting IMF deposition in buffalo

**DOI:** 10.1186/s12864-020-07120-w

**Published:** 2020-10-12

**Authors:** Jieping Huang, Xue Feng, Ruirui Zhu, Duo Guo, Yutong Wei, Xiaodan Cao, Yun Ma, Deshun Shi

**Affiliations:** 1 State Key Laboratory for Conservation and Utilization of Subtropical Agro-Bioresources, Guangxi University, Nanning, 530005 Guangxi China; 2 College of Life Sciences, Xinyang Normal University, Xinyang, 464000 Henan China; 3 School of Agriculture, Ningxia University, Yinchuan, 750021 Ningxia China

**Keywords:** *Bubalus bubalis*, RNA sequencing, Intramuscular fat, Phosphoenolpyruvate carboxykinase 1, Promoter activity

## Abstract

**Background:**

In China, although buffaloes are abundant, beef is mainly obtained from cattle, and this preference is mainly attributed to the low intramuscular fat (IMF) content of buffalo. Genetic factors are an important driver that affects IMF deposition.

**Results:**

To reveal the intrinsic factors responsible for the low IMF content of buffalo, mRNA expression patterns in muscle and adipose tissue between buffalo and cattle were characterized by RNA sequencing analysis. The IMF content in Nanyang cattle was higher than that in Xinyang buffalo. A total of 1566 mRNAs expressed in adipose tissue showed differential expression between the *longissimus dorsi* muscles of buffalo and cattle. Functional annotation suggested a difference in the glycolysis/gluconeogenesis pathway between the two species. The results of RT-qPCR analysis and gain-of-function experiments confirmed the positive association between the IMF content and phosphoenolpyruvate carboxykinase 1 (*PCK1*) expression in buffalo. In both mouse C2C12 cells and cultured bovine myocytes, the activity of the *PCK1* promoter in buffalo is lower than that in cattle. However, in mouse 3T3-L1 adipocytes and cultured bovine adipocytes, the activity of *PCK1* in buffalo promoter is higher than that in cattle.

**Conclusions:**

These results indicate the important role of *PCK1* in buffalo IMF deposition and illustrate the differences between buffalo and cattle promoter activity that drive *PCK1* expression. This research helps to establish a foundation for further studies investigating IMF deposition in buffalo.

## Background

The world population is rapidly growing and will consume two-thirds more animal protein by 2050 relative to today [1]. Thus, the livestock meat industry requires diversified production to ensure stable and sustainable development. Pork, poultry, and beef are the three most heavily consumed meats in the world [2]. In recent years, avian influenza and African swine fever have had significantly negative effects on the meat industry [3, 4]. In contrast, bovines are a reliable and stable source of meat. Cattle meat accounts for the greatest proportion of beef, and other bovine meat, such as buffalo meat, also accounts for a considerable portion of the bovine meat market, especially in several Asian countries [1]. In fact, the nutritional value of buffalo meat is similar to that of cattle meat [5]. Importantly, buffaloes show high disease resistance and adaptability to a wide range of feeding and management conditions [6]. In terms of the population, buffalo is the second largest bovid species next to cattle. Thus, buffalo hold promise for meat production. However, due to the low intramuscular fat (IMF) content, the quality of buffalo meat in terms of flavour and juiciness is poorer than that of cattle meat.

IMF is highly valued because it improves meat quality by enhancing taste, juiciness, and tenderness [7]. IMF deposition has attracted widespread attention for many years. Genetic, managerial, and nutritional factors can affect IMF deposition [8], and genetic factors present moderate or more pronounced effects [9, 10]. For example, in Japanese Black cattle, notably high amounts of IMF (approximately 23%) are deposited in the *longissimus dorsi* muscle (LM), while in European breeds, the IMF content is less than 5% [11]. Comparisons of the gene expression pattern in LM between cattle breeds with high or low IMF content has revealed that many lipid metabolism-related genes are associated with IMF deposition, including adiponectin, C1Q and collagen domain containing (*ADIPOQ*) [12], peroxisome proliferator activated receptor gamma (*PPARγ* or *PPARG*) [13], thyroid hormone responsive [7], and fatty acid binding protein 4 (*FABP4*) [7]. Based on the high-density genotypes, a genome-wide association study suggested that PPARG coactivator 1 alpha, hepatocyte nuclear factor 4 gamma, and forkhead box P3 participated in IMF deposition in cattle [14]. Noncoding RNAs have also been reported to be involved in IMF deposition [15, 16]. Although many studies have been performed on this subject, the molecular mechanism governing IMF deposition has not been elucidated.

In buffaloes, the low IMF content is mainly due to the long-term selection for draught power. Uncovering the genetic differences in the IMF content between buffalo and cattle may provide guiding significance for the improvement of buffalo meat quality. In this study, RNA sequencing was performed to compare mRNA expression patterns in muscle and adipose tissues between buffalo and cattle. A series of analyses revealed that the expression pattern of phosphoenolpyruvate carboxykinase 1 (*PCK1*), a gene with a significant association with IMF, differs between buffalo and cattle in adipose and muscle cells. Overexpression of *PCK1* promotes lipid accumulation in buffalo intramuscular adipocytes. The results of this study suggest a positive role of *PCK1* in buffalo IMF deposition, which can be used as a molecular marker for the improvement of buffalo meat quality through breeding.

## Results

### IMF content of Xinyang buffalo and Nanyang cattle

To evaluate the effects of fattening in Xinyang buffalo and Nanyang cattle, the back fat thickness, LM area (LMA), and IMF content were measured at slaughter. As shown in Table 1, the LMA of cattle was 70.71 ± 11.19 cm^2^, which was larger than that of buffalo (59.61 ± 8.34 cm^2^); the back fat thickness of cattle (4.03 ± 0.81 cm) was higher than that of buffalo (1.03 ± 0.24 cm). Correspondingly, the IMF content in cattle (2.43%) was higher than that in buffalo (0.51%), which could also be observed after slaughter (Additional file 1: Fig. S1).

**Table 1:** Comparison of carcass traits between Xinyang buffalo and Nanyang cattle

Trait	Xinyang buffalo (*n* = 6)	Nanyang cattle (*n* = 6)
loin muscle area (cm^2^)	59.61 ± 8.34	70.71 ± 11.19
back fat thickness (cm)	1.03 ± 0.24	4.03 ± 0.81
IMF content (%)	0.51 ± 0.03	2.34 ± 0.31

### Overview of mRNA expression profiles

**Fig. 1:**
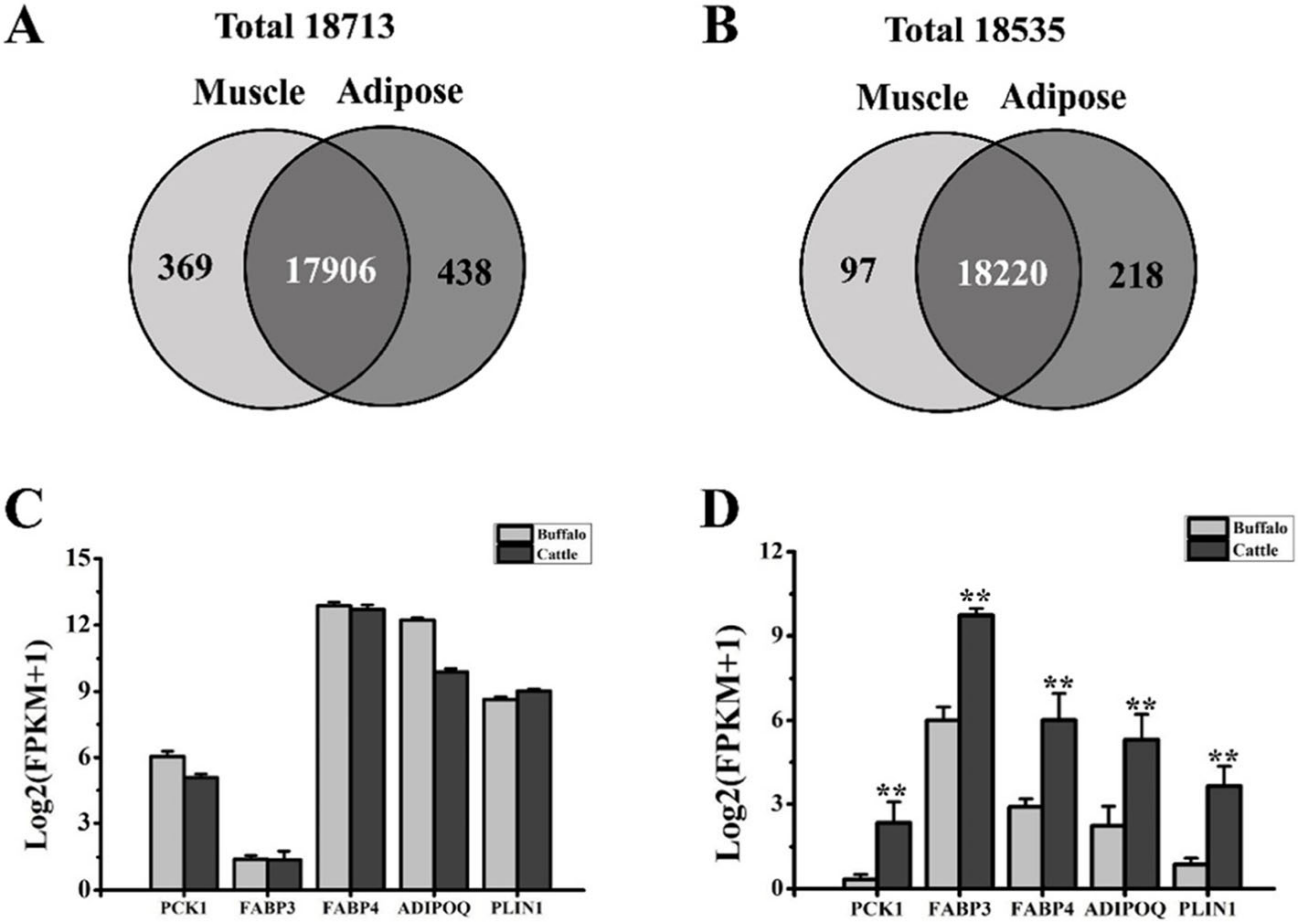
Comparative analysis of mRNA expression between muscle and adipose tissues in buffaloes and cattle based on RNA sequencing. **a** Venn chart for mRNAs expressed in buffalo muscle and adipose tissue. **b** Venn chart for mRNAs expressed in cattle muscle and adipose tissue. Comparison of the expression levels of five adipogenesis markers between buffalo and cattle adipose tissues (**c**) and muscle tissues (**d**). The expressional data are presented as the mean ± S.E. (*n* = 3); ** indicates *p* < 0.01

To compare the gene expression pattern of IMF between buffalo and cattle, RNA sequencing was performed. The IMF in cattle and buffalo was too limited to be sampled for RNA sequencing analysis. Thus, both muscle and adipose tissues were used for analysis in this study. A total of 12 cDNA libraries were constructed, and 12 RNA sequencing datasets were obtained. After a highly stringent filtering pipeline and analyses, 18,713 and 18,535 mRNAs were identified in buffaloes and cattle, respectively (Additional file 2: Table S1 and Additional file 3: Table S2). In buffaloes, 369 mRNAs were specifically expressed in muscle, and 17,906 coexpressed mRNAs were identified in both muscle and adipose tissues (Fig. 1a). In cattle, 97 mRNAs were specifically identified in muscle, and 18,220 coexpressed mRNAs were identified in both tissues (Fig. 1b).

### Differential expression analysis

**Fig. 2:**
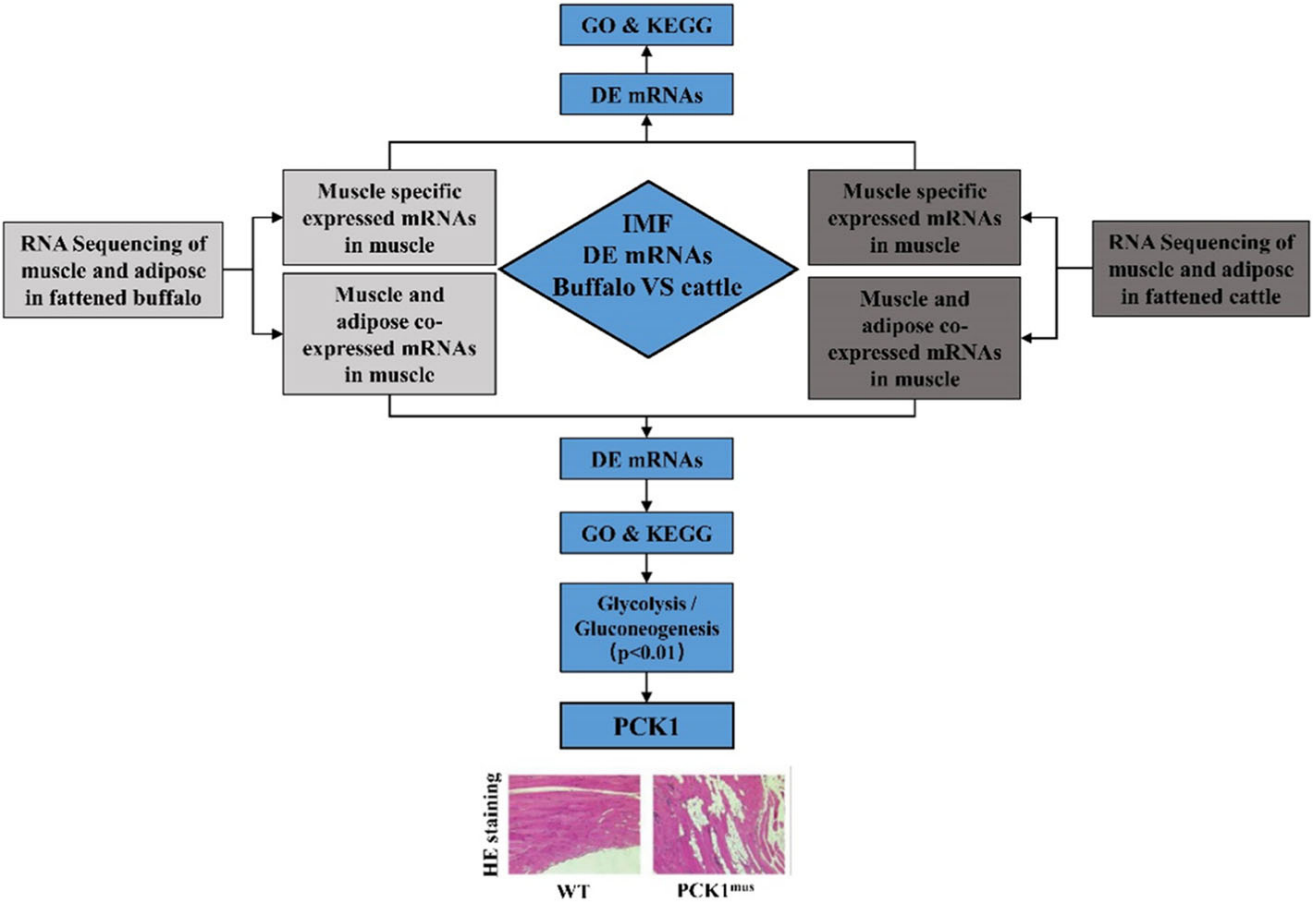
Schematic illustration of RNA sequencing analysis in this study. The results of HE staining are from a previous study [17]

**Fig. 3:**
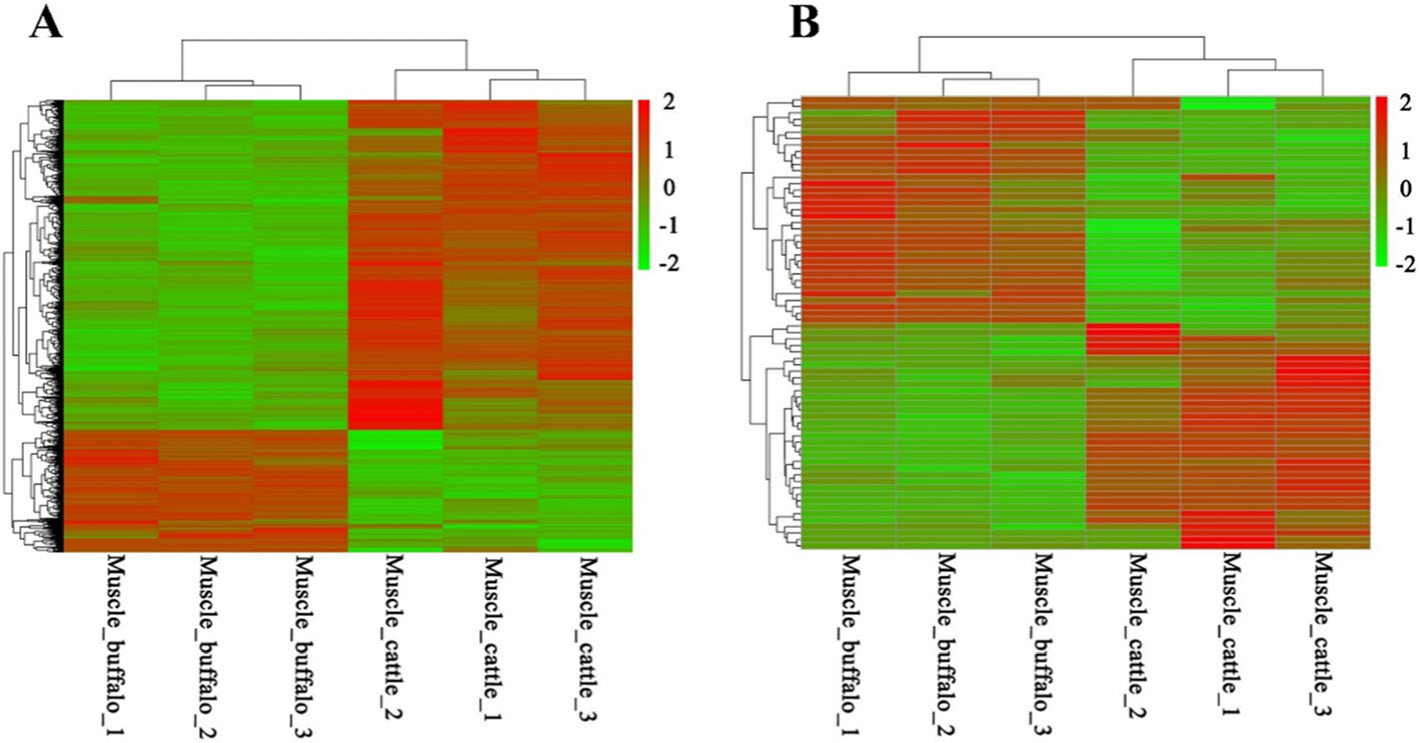
Hierarchical clustering of differentially expressed mRNAs. **a** Hierarchical clustering of 1566 differentially expressed mRNAs (coexpressed in muscle and adipose tissue) in muscle between buffalo and cattle. Hierarchical clustering of 70 differentially expressed mRNAs (specifically expressed in muscle) in muscle between buffalo and cattle

The analysis strategy used to identify intrinsic factors related to the low IMF content in buffaloes is presented in Fig. 2. mRNAs expressed in IMF should be involved in the coexpressed mRNAs of muscle and adipose tissues. In addition, IMF deposition can be affected by the muscle [18, 19]. Therefore, a differential expression analysis was performed with the coexpressed mRNAs and muscle-specific mRNAs between buffalo and cattle (Fig. 2). In total, 1566 differentially expressed (DE) coexpressed mRNAs were ultimately identified, with 1143 upregulated mRNAs and 423 downregulated mRNAs in the muscle of cattle (Fig. 3a and Additional file 4: Table S3). Among these, adipose markers such as *PCK1*, fatty acid binding protein 3 (*FABP3*), *FABP4*, *ADIPOQ*, and perilipin 1 (*PLIN1*) were significantly upregulated in the muscle of cattle (Fig. 1d). In adipose tissue, however, significant differences were not found between buffalo and cattle (Fig. 1c). These results also indicated that the IMF level in cattle was higher than that in buffaloes, which was consistent with the slaughter traits presented in Table 1. In addition, 70 DE muscle-specific mRNAs were identified between buffalo and cattle (Fig. 3b and Additional file 5: Table S4). Interestingly, 35 mRNAs were upregulated and 35 mRNAs were downregulated in cattle. Both hierarchical clustering of the 1566 DE coexpressed mRNAs and 70 DE muscle-specific mRNAs could distinguish buffalo muscle from cattle muscle (Fig. 3), which indicated the high quality of RNA sequencing.

### RT-qPCR validation of differentially expressed mRNAs

**Fig. 4:**
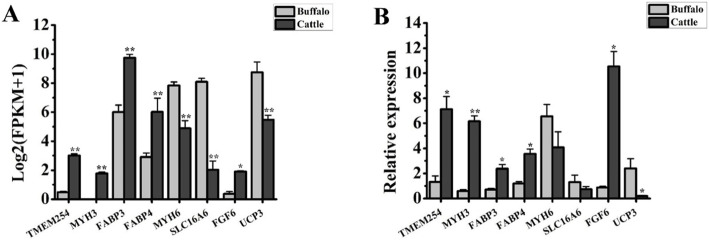
Validation of the differentially expressed mRNAs by RT-qPCR. Expressional patterns of differentially expressed mRNAs analysed by RNA sequencing (**a**) and RT-qPCR (B). *UXT* was used to normalize the expression level of genes, and their prime sequences were conserved between buffalo and cattle. Expression level data are presented as the mean ± S.E. (*n* = 6); * indicates *p* < 0.05; ** indicates *p* < 0.01

To evaluate the quality of the results in the differential expression analysis, 6 DE coexpressed mRNAs and 2 DE muscle-specific mRNAs were randomly selected for validation via RT-qPCR. As shown in Fig. 4, the expression patterns of DE mRNAs in RT-qPCR were approximately similar to those of RNA sequences, although no significant difference was found for *MYH6* and *SLC16A6*. These results indicate the relatively high quality of the differential expression analysis; thus, the DE mRNAs can be used for the following analysis.

### Functional enrichment

To characterize the putative functions of DE mRNAs, functional enrichment was performed for the 1566 DE coexpressed mRNAs and the 70 DE muscle-specific mRNAs. The results showed that the muscle-specific DE mRNAs were enriched in five Gene Ontology (GO) terms, namely, muscle filament sliding, muscle contraction, skeletal muscle contraction, regulation of striated muscle contraction, and sarcomere (Additional file 6: Table S5). Clearly, the terms were all associated with the development of muscle tissue. The aim of our functional enrichment was to find items associated with fat deposition. Thus, we did not investigate these results further.

The coexpressed DE mRNAs were enriched in 41 GO terms and seven Kyoto Encyclopedia of Genes and Genomes (KEGG) pathways (Additional file 7: Table S6). However, only glycolysis/gluconeogenesis (hsa00010), a pathway with a significant effect on fat deposition, was identified (Additional file 7: Table S6). Seventeen DE genes were included in the pathway. Among these genes, *PCK1* has been demonstrated to be associated with fat deposition [20]. Overexpression of *PCK1* in skeletal muscle can improve IMF levels in mice [17]. These results indicate that *PCK1* may be a candidate gene that regulates IMF deposition in buffaloes.

### Expression pattern of PCK1

**Fig. 5:**
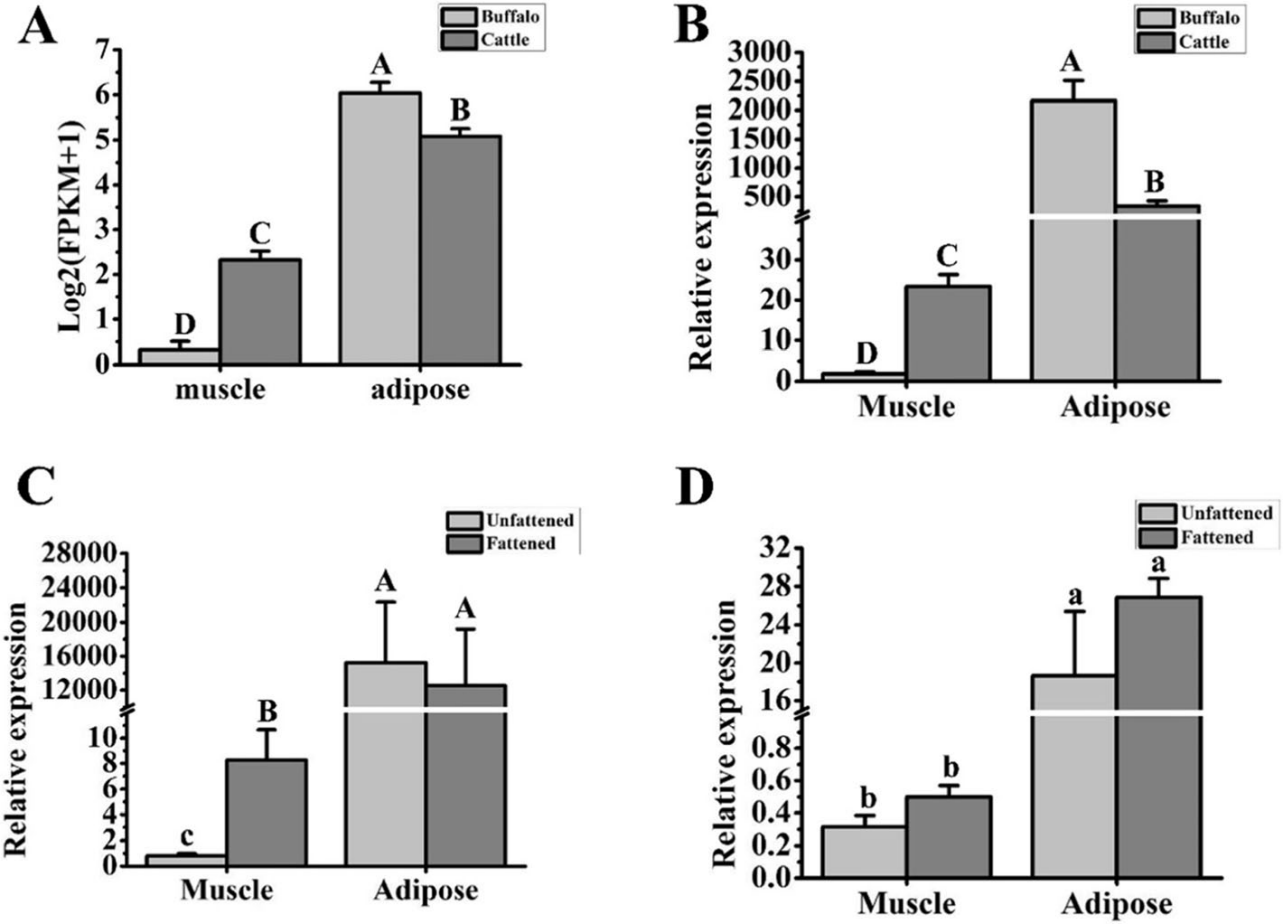
mRNA expression pattern of PCK1 in muscle and adipose tissue of buffalo and cattle. Comparative analysis of mRNA expression levels in adipose and muscle tissue was performed in buffaloes and cattle by RNA sequencing (**a**) and RT-qPCR (**b**). Expression pattern of *PCK1* in adipose and muscle of fattened and unfattened animals in buffaloes (**c**) and in cattle (**d**). *UXT* was used to normalize the expression level of *PCK1,* and their prime sequences were conserved between buffalo and cattle. Expression level data are presented as the mean ± S.E. (RNA sequencing, *n* = 3; RT-qPCR, *n* = 6); lower case indicates *p* < 0.05; upper case indicates *p* < 0.01

According to the RNA sequencing analysis, *PCK1* was upregulated in adipose tissue compared to muscle tissue in both buffalo and cattle (Fig. 5a). In addition, the expression of *PCK1* in cattle muscle was higher than that in buffalo muscle (Fig. 5a). The mRNA expression level of *PCK1* was then validated by RT-qPCR. The sequences of *PCK1* primers were totally conserved between buffalo and cattle. Consistent with the results of RNA sequencing, *PCK1* in adipose tissue was richer than that in muscle tissue in both buffalo and cattle (Fig. 5b, *p* < 0.01). In muscle, the expression of PCK1 was higher in cattle than that in buffalo (Fig. 5b, *p* < 0.01). In addition, compared to unfattened buffalo, the expression of *PCK1* in muscle was upregulated in fattened buffalo (Fig. 5c, *p* < 0.05). These findings indicated a positive correlation between *PCK1* and IMF content in buffalo.

### Effects of *PCK1* on the adipogenic differentiation of buffalo intramuscular preadipocytes

**Fig. 6:**
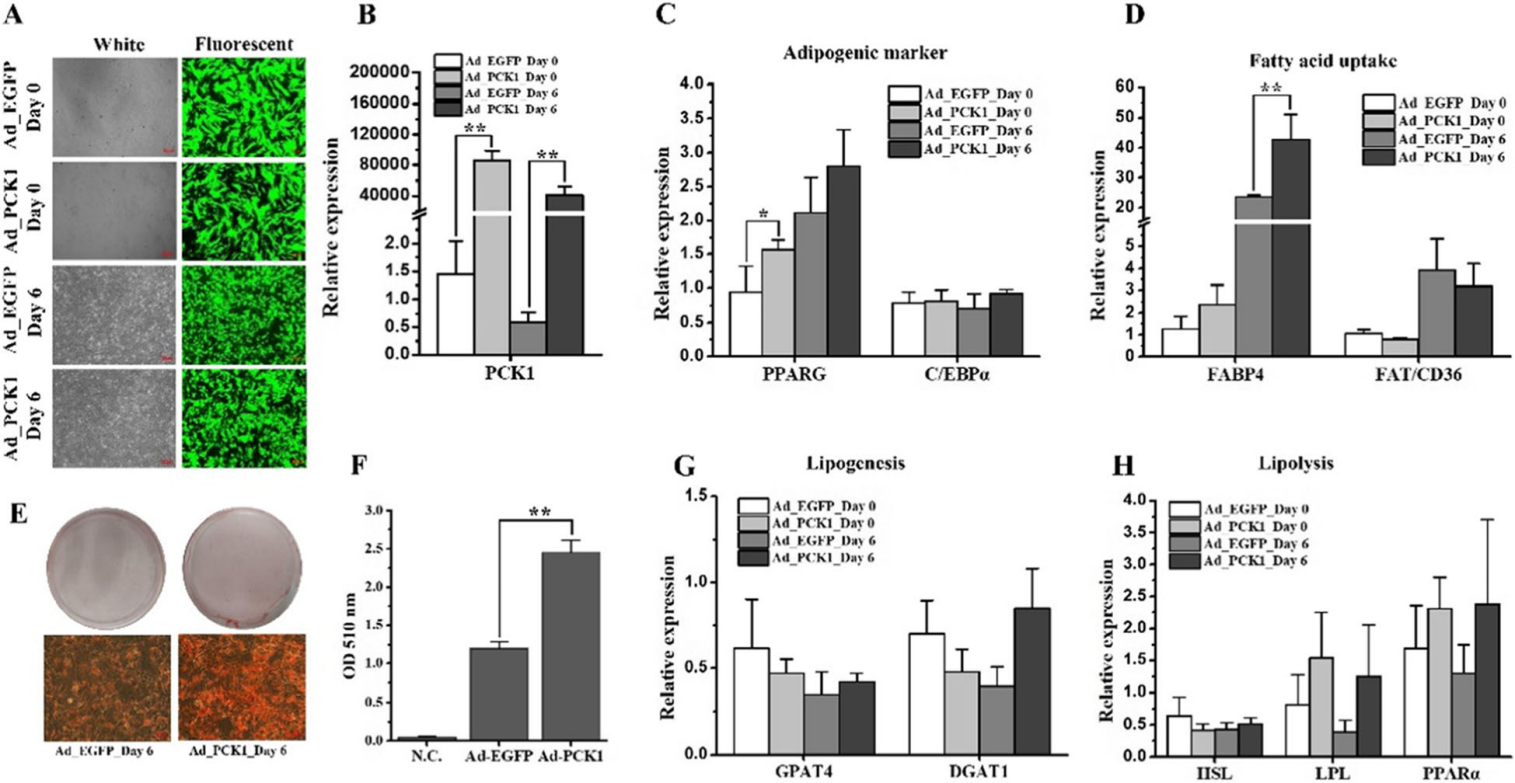
Overexpression of PCK1 promotes lipoid accumulation in buffalo intramuscular adipocytes. **a** Micrographs of EGFP-positive cells under white and fluorescent light in the Ad_EGFP (control) and Ad_PCK1 groups on day o and day 6 of adipogenesis induction. The preadipocytes were induced to differentiation 24 h after adenovirus transduction. Scale bar, 200 μm. **b**-**d**, **g**, **h** The RNA expression levels of *PCK1*, *PPARG*, *C/EBPα*, *FABP4*, *FAT/CD36*, *GPAT4*, *DGAT1*, *HSL*, *LPL*, and *PPARα* on day 0 and day6 of adipogenesis induction in buffalo intramuscular adipocytes transfected with Ad_EGFP and Ad_PCK1. Expression level data are presented as the mean ± S.E. (*n* = 6); * indicates *p* < 0.05; ** indicates *p* < 0.01. **e** Images of Oil Red O staining in buffalo intramuscular adipocytes transfected with Ad_EGFP and Ad_PCK1 on day 6 of adipogenesis induction. Scale bar, 50 μm. **f** Histogram showing the quantitation of Oil Red O staining by spectrophotometry. N.C., negative control by using isopropanol. Data are presented as the mean ± S.E. (*n* = 6); * indicates *p* < 0.05; ** indicates *p* < 0.01

To detect the effect of *PCK1* on the adipogenic differentiation of buffalo intramuscular adipocytes, the CDS region of buffalo *PCK1* was inserted into the pHBAd vector and packaged into adenovirus for overexpression (Ad_PCK1), with EGFP as an internal indicator. Ad_EGFP was used as a control. EGFP was highly expressed on day 0 (24 h after overexpression) and day 6 of adipogenesis induction (Fig. 6a), which indicated a high level of overexpression. Indeed, the expression level of *PCK1* in Ad_PCK1 cells was significantly higher than that in Ad_EGFP cells (Fig. 6b, *p* < 0.01). The adipogenic marker *PPARG* was upregulated with the overexpression of *PCK1* on day 0 of adipogenesis induction (Fig. 6c, *p* < 0.05). On day 6, the fatty acid uptake gene (*FABP4*) was upregulated in Ad_PCK1 group (Fig. 6d, *p* < 0.01). No significant difference was detected for CCAAT enhancer binding protein alpha (*C/EBPα*) (Fig. 6c), fatty acid translocase (*FAT/CD36*) (Fig. 6d), lipogenesis genes (glycerol-3-phosphate acyltransferase 4 (*GPAT4*) and diacylglycerol O-acyltransferase 1 (*DGAT1*)) (Fig. 6g), and lipolysis genes (hormone-sensitive lipase (*HSL*), lipoprotein lipase (*LPL*), and peroxisome proliferator activated receptor alpha (*PPARα*)) (Fig. 6h) between Ad_EGFP and Ad_PCK1 groups. Compared to the Ad_EGFP group, lipid accumulation was significantly enhanced in the Ad_PCK1 group on day 6 of adipogenesis induction (Fig. 6e and f, *p* < 0.01).

### Transcriptional activity of bovine PCK1 promoter

**Fig. 7:**
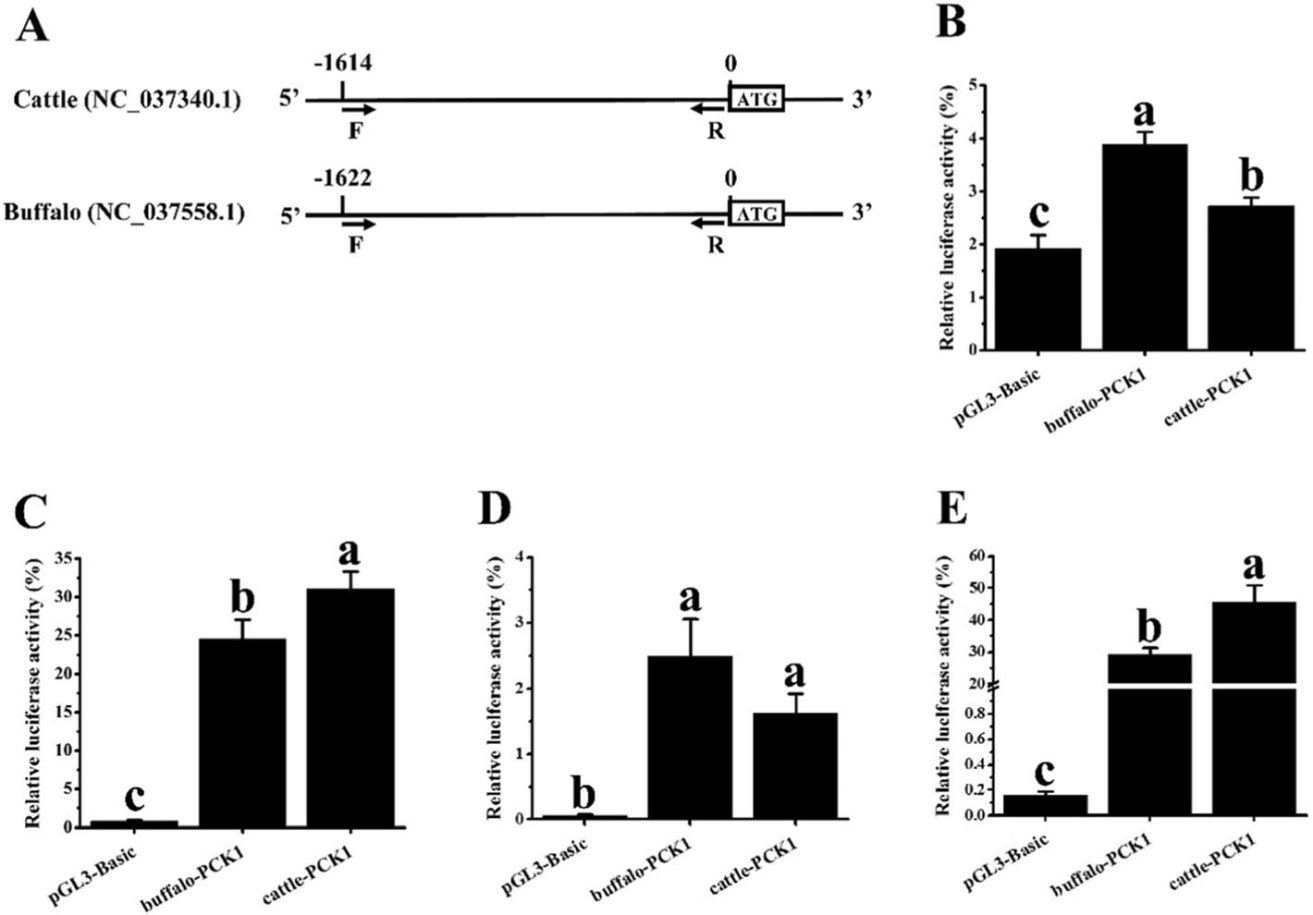
Promoter activity of buffalo and cattle *PCK1* in adipocytes and myoblasts. **a** Putative promoter regions of buffalo and cattle *PCK1* were cloned for activity analysis. Comparison of promoter activity of buffalo and cattle *PCK1* in 3T3-L1 cells (**b**), C2C12 cells (**c**), bovine adipocytes (**d**), and bovine myoblasts (**e**). Data are presented as the mean ± S.E. (*n* = 6); lower case indicates *p* < 0.05; upper case indicates *p* < 0.01

Although the expression pattern of *PCK1* between muscle and adipose tissue in buffaloes was similar to that in cattle, *PCK1* in muscle was more abundant in cattle than in buffaloes, and the opposite finding was observed in adipose tissue (Fig. 5a and 3b). These results suggested that *PCK1* in muscle was more active in cattle; in contrast, *PCK1* in adipose tissue was richer in buffaloes. Then, the transcriptional activity of the *PCK1* promoter of buffalo and cattle was further analysed. The upstream putative promoter of *PCK1* (Fig. 7a) was contained in the luciferase reporter construct and transfected into 3T3-L1 cells, C2C12 cells, primary bovine adipocytes, and primary bovine myoblasts. In 3T3-L1 cells and primary bovine adipocytes, the *PCK1* promoter of buffalo demonstrated higher transcriptional activity than that of cattle (Fig. 7b and d, *p* < 0.05). In C2C12 cells and primary bovine myoblasts, the *PCK1* promoter of cattle presented higher activity than that of buffalo (Fig. 7c and e, *p* < 0.05).

## Discussion

IMF deposition is a highly complex process that can be affected by genetic, managerial, and nutritional factors [8]. Among these factors, genetics is a significant driver. Japanese Black is the best beef breed and exhibits more than 20% IMF content at 24 months of age [11]. By comparison, other European breeds, such as Belgian Blue and German Angus, have only 0.6 and 4.4% IMF content, respectively [11]. IMF levels in Chinese breeds are similar to those of European breeds. In Qinchuan cattle, the IMF content ranges from ~ 3% to ~ 4% at 18 months of age [21]. Nanyang cattle at 24 months of age have an IMF content of (1.88 ± 0.64)% [22]. In the present study, the IMF content of Nanyang cattle at 30 months of age was 2.43% (Table 1). In contrast, Xinyang buffalo had only a 0.51% IMF content (Table 1). Accordingly, the back fat thickness and LMA in Nanyang cattle were higher than those in Xinyang buffalo. Xinyang buffalo and Nanyang cattle were raised with similar forage and feeding management conditions. Thus, the difference in IMF content in animals between the two groups may be attributed to genetic factors.

To identify candidate genes for IMF deposition, a large number of studies have been carried out in cattle [10]. Comparisons of gene expression between breeds with variable IMF contents have indicated that some adipogenic transcription factors are associated with IMF deposition in cattle [7, 12, 13]. Because the IMF content in the tissue was too limited to be sampled for transcriptome analysis or RT-qPCR assay, both muscle (containing very limited amounts of IMF) and adipose tissues were used to identify genes that explain the difference in the IMF content between buffalo and cattle according to the strategy shown in Fig. 2. Simply stated, the coexpressed mRNAs in muscle and adipose tissues were considered to be part of the mRNAs expressed in IMF tissue. Moreover, muscle-specific mRNAs also affect IMF deposition [18, 19]. Both the coexpressed mRNAs and muscle-specific mRNAs that showed differential expression between buffalo and cattle muscle tissues were used for functional enrichment.

The number of mRNAs identified in buffalo muscle and adipose tissue is similar to that of cattle (Fig. 1a and b), which indicates a similar level of complexity between buffalo and cattle meat. However, there were 1566 coexpressed mRNAs (Additional file 4: Table S3) and 70 muscle-specific mRNAs (Additional file 5: Table S4) that showed differential expression between the two bovid species, suggesting a significant difference between buffalo and cattle IMF deposition. Of the 1566 DE mRNAs, 112 and 38 were also identified between Wagyu and Piedmontese [7] and between Japanese Black and Holstein Friesian breeds [23], respectively. However, only four of these DE genes were shared by the three studies, and they showed variable expression patterns among the three studies. These results indicate that the DE genes can be variable from different breed pairs and suggest a highly complex genetic regulation mechanism in IMF deposition.

Based on its specific location, IMF deposition can be affected by both the activity of adipocytes and muscle-specific factors. Coculturing C2C12 and 3T3-L1 preadipocyte cells downregulates adipogenic marker gene expression [18]. Myostatin, which is secreted by myoblasts, decreases IMF deposition by inhibiting the differentiation of intramuscular preadipocytes [19]. However, a similar factor that is responsible for the difference in IMF deposition between buffalo and cattle was not identified in this study; all 5 identified GO items with Benjamini values (*p*) < 0.05 were associated with muscle development (Additional file 6: Table S5).

Lipid metabolism is notably different between buffalo and cattle, which is also reflected by the difference in milk fat content [24, 25]. The percentage of milk fat in buffaloes is higher than that in cattle [24, 26, 27]. A previous study suggested that four genes (*KCTD8*, *FOXO4*, *SSTR3*, and *LOC782855*) related to carbohydrate metabolism and a gene (*ESRRG*) related to lipid metabolism may contribute to the differences between buffalo and cattle milk fat content [25]. In the present study, however, these five genes did not show differential expression between buffalo and cattle muscle tissues, and no item marked with “carbohydrate metabolism” was identified in our functional enrichment analysis. Instead, an energy metabolism pathway, the glycolysis/gluconeogenesis pathway (*p* < 0.01), was identified by the functional enrichment of the DE coexpressed mRNAs (Fig. 2; Additional file 7: Table S6). Glycolysis is a catabolic pathway that reverses the carbon flow of anabolic pathways, gluconeogenesis and glyceroneogenesis [28]. Essentially, glyceroneogenesis is an abbreviated version of gluconeogenesis responsible for the de novo synthesis of glycerol-3-phosphate from the precursors of gluconeogenesis [29]. Glycerol-3-phosphate can esterify fatty acyl-CoAs into triglycerides (TGs). Thus, the carbon for TG comes from glycolysis. The hyperactivity of glyceroneogenesis enhances the storage of TG and results in fat deposition in muscle [30].

A total of 17 DE genes were involved in the glycolysis/gluconeogenesis pathway. Among them, *PCK1* drives a significant and irreversible reaction that is shared by glyceroneogenesis and gluconeogenesis [28]. In fact, *PCK1* is considered an adipogenic marker, an obesity gene, and even an IMF deposition-related gene [17, 31]. In mice, white adipose tissue-specific overexpression of *PCK1* increases the rate of glyceroneogenesis in adipose tissue and results in obesity [32]. In contrast, knockdown of *PCK1* expression in adipose tissue results in lipodystrophy in mice [20]. In skeletal muscle tissue, specific overexpression of *PCK1* enhances IMF deposition in mice [17]. In pigs, the expression level of *PCK1* demonstrates a positive association with the IMF content and fatty acid composition [31]. Functional mutation of the *PCK1* gene results in a lower IMF content in pigs [33]. Interestingly, pigs with *PCK1* specifically expressed in skeletal muscle show an enhanced IMF content [34]. In cattle, *PCK1* is upregulated in Wagyu breed (high IMF) compared to Piedmontese breed (low IMF) [7]. In the present study, *PCK1* was downregulated in buffalo muscle tissue (Fig. 5a and b), which may be responsible for the lower IMF content in buffaloes (Table 1). Interestingly, overexpression of *PCK1* promoted the expression of the adipogenic marker *PPARG* (Fig. 6c, *p* < 0.05) and the lipid accumulation in buffalo intramuscular adipocytes (Fig. 6e and f, *p* < 0.01), suggesting a positive role of *PCK1* in IMF deposition in buffalo. The expression level of fatty acid uptake gene *FABP4* was upregulated by *PCK1* on day 6 (Fig. 6d, *p* < 0.01). FABP4 is a fatty acid binding protein and promotes fatty acid transport [35]. As mentioned above, PCK1 drives glyceroneogenesis to produce glycerol-3-phosphate [28]. Both fatty acid and glycerol-3-phosphate can be utilized for TGs synthesis [28, 29]. Therefore, overexpression of *PCK1* enhances lipid accumulation by upregulating the expression of *PPARG* and *FABP4* in buffalo intramuscular adipocytes, which is consistent with the effects of *PCK1* in other species (20,35).

We hypothesized that there should be some intrinsic reasons for the lower expression of *PCK1* in buffalo muscle than in cattle muscle. In terms of the *PCK1* putative promoter sequence, differences can be found between buffalo and cattle, with 44 single-base, 2 double-base, and 1 tribasic base mutations (Additional file 8: Fig. S2). Accordingly, the activity of the *PCK1* promoter demonstrated variable levels between buffalo and cattle (Fig. 5). Interestingly, the activity of the buffalo *PCK1* promoter is higher than that of cattle in adipocytes, while the opposite occurs in myoblasts (Fig. 5). *PCK1* is widely expressed in multiple tissues and has high levels in the liver and adipose tissue [36], which also suggests its important role in lipid metabolism. The molecular mechanism driving *PCK1* expression is highly complex, and its expression in different tissues can be regulated in a variable manner according to its specific role in the tissue. The promoter of *PCK1* has been widely studied, and some significant transcription factors have been identified, such as *PPARG*, sterol regulatory element binding protein 1, and CCAAT/enhancer binding protein beta [36]. Recently, a large number of noncoding RNAs have been demonstrated to participate in the regulation of coding genes via interaction with promoters [37, 38]. In addition, methylation in the promoter sequence can influence the expression of a gene [39]. Therefore, the underlying molecular mechanism driving the expression of *PCK1* in the promoter regions of buffalo and cattle, such as DNA methylation, protein-DNA interactions, and RNA-DNA interactions, needs further study.

## Conclusions

This study evaluated the IMF content of fattened buffalo and cattle and compared the mRNA expression patterns in muscle and adipose tissue between the two bovid species. This study demonstrates that (1) the IMF content of fattened cattle at 30 months of age is higher than that of fattened buffalo, as well as other relative traits, including back fat thickness and LMA; (2) a large number of mRNAs have differential expression between buffalo and cattle muscles; (3) the glycolysis/gluconeogenesis pathway may regulate IMF deposition in buffalo; (4) *PCK1* shows a positive association with the IMF content in buffalo; and (5) *PCK1* promoter activity of buffalo is higher than that of cattle in adipocytes, which is in contrast to that of myoblasts.

## Methods

The IMF content in buffalo is lower than that in cattle, and a genetic factor should be responsible for such a difference. The aim of this research is to identify the genetic factors responsible for the low IMF content in buffalo. Because the IMF content in tissue is too limited to be sampled for detection, both muscle and adipose tissues were used for high-throughput RNA sequencing and further validation.

### Animals, sample collection, and fattening traits

Xinyang buffaloes (*n* = 12) and Nanyang cattle (*n* = 12) were raised in the breeding farm of Xinyang Buffalo (Guangshan, Henan Province, P.R. China) and the breeding centre of Nanyang cattle (Nanyang, Henan Province, P.R. China), respectively, with equivalent forage and feeding management conditions. They were weaned at 3 months of age, castrated at 6 months of age, started to fatten at 18 months of age, and slaughtered at 30 months of age. Details of the feeding conditions were described in a previous study [40]. In this study, only six buffaloes and six cattle were fattened, and the others were not fattened. For the unfattened animals, concentrate was replaced by roughage from 18 to 30 months of age.

The LM (muscle) and back subcutaneous fat (adipose) tissues were sampled immediately after slaughter. For determination of IMF, fresh muscle was kept in an icebox and taken back to the laboratory immediately. For RNA extraction, tissue was frozen immediately in liquid nitrogen and stored at − 80 °C.

The back fat thickness, LMA, and IMF content of fattened animals were measured. The 12th–13th rib back fat thickness and LMA were measured by an Ultrasonic Backfat Tester (HS-1600 V; Honda Electronics, Toyohashi, Japan) at slaughter. The IMF content was measured by a near-infrared meat quality analyser (Series 3000 Food Analyser, NIR, Australia).

### RNA isolation and sequencing

Total RNA was extracted using TRIzol reagent (Invitrogen, USA) according to the manufacturer’s instructions. The quality and concentration of total RNA were determined by a NanoDrop 2000 (Nanodrop, Wilmington, DE) and 1% agarose gel. Total RNA from muscle and adipose tissues (three buffaloes and three cattle) of high quality was sent to Genergy Biotechnology Co., Ltd. (Shanghai, China) for library construction and RNA sequencing. Briefly, approximately 2 μg of total RNA per sample was used for library construction according to the protocol of the TruSeq® RNA LT Sample Prep Kit v2 (Illumina, USA). The cDNA library was sequenced using the double terminal sequencing mode of the Illumina HiSeq 3000 platform. A total of four groups that included the LM and the back fat tissues of buffalo and cattle were used for RNA sequencing. Three samples were obtained for each group.

### Bioinformatics analysis of sequencing data

Low-quality reads and those containing adapters were removed to obtain clean reads using Trim Galore (Version 0.4.2, Babraham Institute, Cambridge, UK) [41]. High-quality data were used for the subsequent analysis. Since annotation information of the buffalo genome was not available, the cattle genome (UMD3.1) was used in the present study. Cattle genome and gene model annotation files were downloaded from the Ensemble database (http://www.ensembl.org/index.html). Reads were aligned to the reference genome using STAR [42]. Mapped reads were assembled using Cufflinks v2.2.1 [43]. These assemblies were merged to the reference gene model annotation by Cuffmerge [44]. Fragments per kilobase of transcript per million mapped reads (FPKM) was used as an index to calculate the expression level of each transcript in every library using the Cuffdiff program. The expression level was presented as log2(FPKM+ 1). The DE mRNAs were identified by DESeq2, with an absolute value of log2(fold change) ≥ 1 and an FDR ≤ 0.05 [44]. DE mRNAs were presented in heatmaps by the R package [45]. Functional enrichment for DE mRNAs was performed using DAVID bioinformatics resources (http://david.abcc.ncifcrf.gov/) [46]. The human database was used. GO terms (including molecular function (MF), biological process (BP), and cellular components (CC)) and KEGG pathways with a Benjamini value (*p*) < 0.05 were considered to be significantly enriched.

### RT-qPCR

Primers were designed by Primer-BLAST (http://www.ncbi.nlm.nih.gov/tools/primer-blast/) (Table 2). The ubiquitously expressed prefoldin-like chaperone (*UXT*) gene was used to normalize the expression level of the candidate gene [47, 48]. Total RNA was transcribed into cDNA using the PrimeScript RT Reagent Kit with gDNA Eraser (TaKaRa, Dalian, China). qPCR was performed using SYBR Green I (TaKaRa, Dalian, China) with two-step reactions according to the manufacturer’s recommended protocol. The efficiency of amplification was detected for each primer pair to ensure a reliable result. The cycle threshold (2^–ΔΔCt^) method was used to calculate the relative expression level of candidate genes. The RT-qPCR analysis was performed with six biological repeats, and each sample had three technological repeats.

**Table 2:** Details of primers used in this study

Name	Sense primer (5′–3′)	Antisense primer (5′–3′)	Size (bp)	Use for
*TMEM254*	CGTGCATGCTTGGTATTGGC	GGAACCAGAGCAGTTGGGTC	116	qPCR
*MYH3*	GAGGTCACATCCCATCTGCTC	CTACTCATGGTGTTGGCTGAGA	109	qPCR
*FABP3*	CGTCTTTCCCAACCTAGCCC	AACCGACACCGAGTGACTTC	111	qPCR
*FABP4*	TTTGCTACCAGGAAAGTGGCT	TGACACATTCCAGCACCATCTT	277	qPCR
*MYH6*	TGTCGGGGACAGCAGTAAAG	TGTTCAGATTCTCCCGGTGG	89	qPCR
*SLC16A6*	TCTTAACATTCACAGCCCCGC	CGATGGCGATGTACATGTGG	147	qPCR
*FGF6*	CCCTACAGCCTGCTGGAGATTT	GGCAGGAGGGTCTCTCTGAA	155	qPCR
*UCP3*	CGACTCCGTCAAGCAGTTCT	TGGGCAGAATTCCTTTCCAC	254	qPCR
*PPARG*	GCAAAGTGGAGCCTGTATC	GTAGTGGAACCCTGACGC	137	qPCR
*C/EBPα*	TGGACAAGAACAGCAACGAG	TTGTCACTGGTCAGCTCCAG	130	qPCR
*FABP4*	AAGTCAAGAGCATCGTAA	CCAGCACCATCTTATCAT	111	qPCR
*FAT/CD36*	TGGAAAGGACGACATAAGCAAA	TGGAAATGAGGCTGCATCTGT	118	qPCR
*GPAT4*	GGGCTTGGGCGTACTCATC	CAGGAACTCCTTGAACCTCCC	136	qPCR
*DGAT1*	GGTCGCGGCCTTCGAT	TCTACGTCTCCGTCCTTGTCT	118	qPCR
*HSL*	CAGTGTCCAAGACAGAGCCA	GCAGCTTCAGGCTTTTGAGG	102	qPCR
*LPL*	GGCAGCGTGAGTAGGACC	CGGCTTTATCAACTCTATTCT	147	qPCR
*PPARα*	GCTCCGTTATTACAGACACCC	AACCCTTGCAGCCCTCAC	180	qPCR
*UXT*	CCGGAAGCCACGGTTCTTA	AGAGATGAAAGCCTCGTAGCG	133	qPCR
*PCK1*-q	GCGGAGTACAAGGGCAAAGT	CAGGTACTGGCCGAAGTTGT	81	qPCR
*PCK1*-V	CGGggtaccAGCCAGCAGTTCCATAGCTC	CCGctcgagGCACGGATGCCGAGGTT	1610	Vector construction

The restriction sites of primers are shown with lowercase characters

### Adenovirus packaging

The overexpression adenovirus packaging was performed by the Hanbio Biotechnology Co.; Ltd. (Shanghai, China) as previously described [40]. Briefly, the CDS of buffalo *PCK1* was synthesized and ligated to the AdMax system. EGFP was included to indicate the transduction efficiency. Ad_EGFP was used as a negative control.

### Isolation of buffalo intramuscular preadipocytes and cell culture

Buffalo intramuscular preadipocytes were isolated as described in a previous study [49]. Briefly, fresh LM tissue was sampled and the visible connective tissue was removed. Muscle tissue was finely minced and digested with 0.2% collagenase type II (Gibco, Grand Island, NY) in a shaking water bath at 37 °C until complete digestion. The digested sample was filtered by 80 μm nylon mesh filters and washed three times with Dulbecco’s modified Eagle’s medium (DMEM) (Gibco, Grand Island, NY). Cells were then cultured with complete culture medium (DMEM with 10% foetal bovine serum and 1% penicillin–streptomycin (Gibco, Grand Island, NY)) under conditions of 37 °C with 5% CO_2_ for 1 h. The complete culture medium was changed to remove the non-adherent cells. The adherent cells (intramuscular preadipocytes) were continuously cultured with complete culture medium. When reaching 90% confluence, the cells were digested and collected for further culture.

### Adenoviral transduction, adipogenic differentiation, oil red O staining, and quantification

The intramuscular preadipocytes were planted in twelve-well plates. Adenoviral transduction was conducted when the cells reached 80% confluence. Two days after transduction, the cells were induced to adipogenic differentiation. Six days later, Oil Red O staining was performed. The Oil Red O was eluted by isopropanol to quantify the lipid accumulation in cells. In addition, cells induced to differentiation for 6 days were collected for RT-qPCR analysis. Details of adenoviral transduction, adipogenic differentiation, Oil Red O staining, and quantification can be found in a previous study [40]. All these experiments were performed with six biological repeats.

### Vector construction

The pGL3-Basic system (Promega, Madison, USA) was used for the promoter activity analysis of *PCK1* as previously described [50]. The putative upstream promoter regions were cloned from the genomic DNA of cattle and buffalo (Fig. 7a and Additional file 8: Fig. S2). Fragments were inserted into the *KpnI* and *XhoI* restriction sites of the pGL3-Basic vector to obtain recombinant plasmids (pGL3-cattle-PCK1 and pGL3-buffalo-PCK1). The pLR-TK vector was used as an internal control.

### Cell culture, transfection, and luciferase reporter assays

3T3-L1 cells (ATCC, Shanghai, China), C2C12 cells (ATCC, Shanghai, China), primary bovine adipocytes, and primary bovine myoblasts were used to detect the activity of putative promoters. Isolation and culture of primary bovine adipocytes and primary bovine myoblasts were performed as previously described [40, 51]. Cells were cultured with complete culture medium in 5% CO_2_ at 37 °C. Cells were plated in a 48-well plate at a density of 1 × 10^6^ cells/well 24 h prior to transfection. Cells were cotransfected with 400 ng recombinant plasmid DNA and 16 ng pLR-TK plasmid using Lipofectamine® 3000 Reagent (Invitrogen, USA) according to the manufacturer’s protocol. After transfection for 48 h, cells were harvested to analyse the activity of each fragment using a Dual-Luciferase Reporter Gene Assay System (Promega, Madison, USA) on a GloMax™ 96 Microplate Luminometer (Promega, Madison, USA). The ratio of the firefly luciferase value to Renilla luciferase value was used to evaluate the activity of the *PCK1* promoter fragment. Each promoter fragment plasmid was analysed with six repeats, and the assay was repeated three times.

### Statistical analysis

Data were analysed by SPSS 19 software. Significant difference analysis was performed with one-way ANOVA. A *p*-value < 0.05 was considered to indicate significant differences. The results are presented as the means ± standard error (S.E.) using the OriginPro 8.5 program.

## Supplementary information

**Additional file 1: Fig. S1.** Images of the cross-section of the *longissimus dorsi* muscle in the 12th–13th rib of buffalo (A) and cattle (B). Intramuscular fat (IMF) of the *longissimus dorsi* muscle is very limited in both buffalo and cattle. IMF in cattle is slightly richer than that in buffaloes. Tissue in the yellow box was sampled for the experiment.

**Additional file 2: Table S1.** Total mRNAs expressed in the *longissimus dorsi* muscle and adipose tissue of buffalo.

**Additional file 3: Table S2.** Total mRNAs expressed in the *longissimus dorsi* muscle and adipose tissue of cattle.

**Additional file 4: Table S3.** Differentially expressed genes between buffalo and cattle in coexpressed mRNAs.

**Additional file 5: Table S4.** Differentially expressed genes between buffalo and cattle in the *longissimus dorsi* muscle specific mRNAs.

**Additional file 6: Table S5.** Functional enrichment of differentially expressed genes in the *longissimus dorsi* muscle-specific mRNAs.

**Additional file 7: Table S6.** Functional enrichment of differentially expressed genes in coexpressed mRNAs.

**Additional file 8: Fig. S2.** Sequences of the upstream region of the buffalo and cattle *PCK1* genes. * under sequences indicates base difference between buffalo and cattle. Horizontal lines under sequence indicate the forward primer (F), the reverse primer (R), and the start codon (ATG), respectively.

## Data Availability

RNA sequencing data were deposited in the Genome Expression Omnibus of NCBI. Accession number of three buffalo adipose tissues is GSE112744 and that of others is GSE139102.

## Abbreviations

IMF: Intramuscular fat
PCK1: Phosphoenolpyruvate carboxykinase 1
LM: *Longissimus dorsi* muscle
LMA: LM area
ADIPOQ: Adiponectin, C1Q and collagen domain containing
PPARγ or PPARG: Peroxisome proliferator activated receptor gamma
FABP3: Fatty acid binding protein 3
FABP4: Fatty acid binding protein 4
PLIN1: Perilipin 1
MYH6: Myosin heavy chain 6
SLC16A6: Solute carrier family 16 member 6
GO: Gene Ontology
KEGG: Kyoto Encyclopedia of Genes and Genomes
C/EBPα: CCAAT enhancer binding protein alpha
FAT/CD36: Fatty acid translocase
GPAT4: Glycerol-3-phosphate acyltransferase 4
DGAT1: Diacylglycerol O-acyltransferase 1
HSL: Hormone-sensitive lipase
LPL: Lipoprotein lipase
PPARα: Peroxisome proliferator activated receptor alpha
TG: Triglyceride
FPKM: Fragments per kilobase of transcript per million mapped reads
DE: Differentially expressed
UXT: Ubiquitously expressed prefoldin like chaperone
DMEM: Dulbecco’s modified Eagle’s medium
S.E.: Standard error

## References

[1] Naveena BM, Kiran M (2014). Buffalo meat quality, composition, and processing characteristics: contribution to the global economy and nutritional security. Anim Front.

[2] FAO, 2013. http://www.fao.org/faostat/en/#data.

[3] Su S, Bi Y, Wong G, Gray GC, Gao GF, Li S (2015). The epidemiology, evolution and recent outbreaks of avian influenza viruses in China: a review. J Virol.

[4] Sánchez-Cordón PJ, Montoya M, Reis AL, Dixon LK (2018). African swine fever: a re-emerging viral disease threatening the global pig industry. Vet J.

[5] Kandeepan G, Mendiratta SK, Shukla V, Vishnuraj MR (2013). Processing characteristics of buffalo meat-a review. J Meat Sci Technol.

[6] Wanapat M, Kang SC (2013). World buffalo production: challenges in meat and milk production, and mitigation of methane emission. Buffalo Bull.

[7] Hudson NJ, Reverter A, Greenwood PL, Guo B, Café LM, Dalrymple BP (2015). Longitudinal muscle gene expression patterns associated with differential intramuscular fat in cattle. Animal.

[8] Park SJ, Beak SH, Jung DJS, Kim SY, Jeong IH, Piao MY (2018). Genetic, management, and nutritional factors affecting intramuscular fat deposition in beef cattle. Asian-Australas J Anim Sci.

[9] Bouchard C (2010). Genetic factors in the regulation of adipose tissue distribution. Acta Medica Scand Suppl.

[10] Campos CF, Duarte MS, Guimarães SE, Verardo LL, Wei S, Du M (2016). Review: animal model and the current understanding of molecule dynamics of adipogenesis. Animal..

[11] Gotoh T, Albrecht E, Teuscher F, Kawabata K, Sakashita K, Iwamoto H (2009). Differences in muscle and fat accretion in Japanese black and European cattle. Meat Sci.

[12] Wang YH, Bower NI, Reverter A, Tan SH, De Jager N, Wang R (2009). Gene expression patterns during intramuscular fat development in cattle. J Anim Sci.

[13] De Jager N, Hudson NJ, Reverter A, Barnard R, Café LM, Greenwood PL (2013). Gene expression phenotypes for lipid metabolism and intramuscular fat in skeletal muscle of cattle. J Anim Sci.

[14] Ramayo-Caldas Y, Fortes MR, Hudson NJ, Porto-Neto LR, Bolormaa S, Barendse W (2014). A marker derived gene network reveals the regulatory role of PPARGC1A, HNF4G and FOXP3 in intramuscular fat deposition of beef cattle. J Anim Sci.

[15] Chen FF, Xiong Y, Peng Y, Gao Y, Qin J, Chu GY (2017). mir-425-5p inhibits differentiation and proliferation in porcine intramuscular preadipocytes. Int J Mol Sci.

[16] Huang J, Wang S, Feng X, Liu X, Zhao J, Zheng Q (2019). miRNA transcriptome comparison between muscle and adipose tissues indicates potential miRNAs associated with intramuscular fat in Chinese swamp buffalo. Genome.

[17] Hakimi P, Yang J, Casadesus G, Massillon D, Tolentino-Silva F, Nye CK (2007). Overexpression of the cytosolic form of phosphoenolpyruvate carboxykinase (GTP) in skeletal muscle repatterns energy metabolism in the mouse. J Biol Chem.

[18] Muthuraman P (2014). Effect of coculturing on the myogenic and adipogenic marker gene expression. Appl Biochem Biotechnol.

[19] Komolka K, Albrecht E, Wimmers K, Michal JJ, Maak S (2014). Molecular heterogeneities of adipose depots-potential effects on adipose-muscle cross-talk in humans, mice and farm animals. J Genomics.

[20] Olswang Y, Cohen H, Papo O, Cassuto H, Croniger CM, Hakimi P (2002). A mutation in the peroxisome proliferator-activated receptor γ-binding site in the gene for the cytosolic form of phosphoenolpyruvate carboxykinase reduces adipose tissue size and fat content in mice. PNAS..

[21] Zhang Y, Wang H, Wang Y, Wang HC, Zhang S, Hong JY (2017). Transcriptome analysis of mRNA and microRNAs in intramuscular fat tissues of castrated and intact male Chinese Qinchuan cattle. PLoS One.

[22] Qi Y, Zhang X, Wang Y, Wang D, Guo Z, Liu P (2014). The expression of ADAMTS2 and collagen genes in muscle tissue and its relationship with meat quality characters in cattle. J Yunnan Agric Univ (Natural Science).

[23] Albrecht E, Schering L, Liu Y, Komolka K, Kühn C, Wimmers K (2017). TRIENNIAL GROWTH AND DEVELOPMENT SYMPOSIUM: factors influencing bovine intramuscular adipose tissue development and cellularity. J Anim Sci.

[24] Aspilcueta-Borquis RR, Di Palo R, Araujo Neto FR, Baldi F, de Camargo GMF, de Albuquerque LG (2010). Genetic parameter estimates for buffalo milk yield, milk quality and mozzarella production and Bayesian inference analysis of their relationships. Genet Mol Res.

[25] de Camargo GM, Aspilcueta-Borquis RR, Fortes MR, Porto-Neto R, Cardoso DF, Santos DJ (2015). Prospecting major genes in dairy buffaloes. BMC Genomics.

[26] Cui X, Hou Y, Yang S, Xie Y, Zhang S, Zhang Y (2014). Transcriptional profiling of mammary gland in Holstein cows with extremely different milk protein and fat percentage using RNA sequencing. BMC Genomics.

[27] Bernabucci U, Biffani S, Buggiotti L, Vitali A, Lacetera N, Nardone A (2014). The effects of heat stress in Italian Holstein dairy cattle. J Dairy Sci.

[28] Beale EG, Hammer RE, Antoine B, Forest C (2004). Disregulated glyceroneogenesis: PCK1 as a candidate diabetes and obesity gene. Trends Endocrinol Metab.

[29] Reshef L, Olswang Y, Cassuto H, Blum B, Croniger CM, Kalhan SC (2003). Glyceroneogenesis and the triglyceride/fatty acid cycle. J Biol Chem.

[30] Forest C, Tordjman J, Glorian M, Duplus E, Chauvet G, Quette J (2003). Fatty acid recycling in adipocytes: a role for glyceroneogenesis and phosphoenolpyruvate carboxykinase. Biochem Soc Trans.

[31] Wang W, Xue W, Jin B, Zhang X, Ma F, Xu X (2013). Candidate gene expression affects intramuscular fat content and fatty acid composition in pigs. J Appl Genet.

[32] Franckhauser S, Munoz S, Pujol A, Casellas A, Riu E, Otaegui P (2002). Increased fatty acid re-esterification by PEPCK overexpression in adipose tissue leads to obesity without insulin resistance. Diabetes..

[33] Latorre P, Burgos C, Hidalgo J, Varona L, Carrodeguas JA, López-Buesa P (2016). c.A2456C-substitution in Pck1 changes the enzyme kinetic and functional properties modifying fat distribution in pigs. Sci Rep.

[34] Ren Z, Wang Y, Ren Y, Zhang Z, Gu W, Wu Z (2017). Enhancement of porcine intramuscular fat content by overexpression of the cytosolic form of phosphoenolpyruvate carboxykinase in skeletal muscle. Sci Rep.

[35] Maeda K, Cao H, Kono K, Gorgun CZ, Furuhashi M, Uysal KT (2005). Adipocyte/macrophage fatty acid binding proteins control integrated metabolic responses in obesity and diabetes. Cell Metab.

[36] Chakravarty K, Cassuto H, Reshef L, Hanson RW (2005). Factors that control the tissue-specific transcription of the gene for phosphoenolpyruvate carboxykinase-C. Crit Rev Biochem Mol Biol.

[37] Pian L, Xue W, Kang L, Li Z, Nie Y, Du Z (2018). Targeting the IGF1R pathway in breast cancer using antisense lncRNA-mediated promoter cis competition. Mol Ther Nucleic Acids.

[38] Chen L, Zhi Z, Wang L, Zhao YY, Deng M, Liu YH (2019). NSD2 circular RNA promotes metastasis of colorectal cancer by targeting miR-199b-5p-mediated DDR1 and JAG1 signalling. J Pathol.

[39] Métivier R, Gallais R, Tiffoche C, Le Péron C, Jurkowska RZ, Carmouche RP (2008). Cyclical DNA methylation of a transcriptionally active promoter. Nature..

[40] Huang J, Zheng Q, Wang S, Wei X, Fen L, Yun M (2019). High-throughput RNA sequencing reveals NDUFC2-AS lncRNA promotes adipogenic differentiation in Chinese buffalo (Bubalus bubalis L). Genes..

[41] Krueger F. Trim Galore. http://www.bioinformatics.babraham.ac.uk/projects/trim_galore/.

[42] Dobin A, Davis CA, Schlesinger F, Drenkow J, Zaleski C, Jha S (2013). STAR: ultrafast universal RNA-seq aligner. Bioinformatics..

[43] Trapnell C, Roberts A, Goff L, Pertea G, Kim D, Kelley DR (2012). Differential gene and transcript expression analysis of RNA-seq experiments with TopHat and cufflinks. Nat Protoc.

[44] Anders S, Huber W (2010). Differential expression analysis for sequence count data. Genome Biol.

[45] Heatplus: Heatmaps with row and/or column covariates and colored clusters. R package version 2.1.0. http://www.bioconductor.org/packages/2.10/bioc/html/Heatplus.html.

[46] Huang DW, Sherman BT, Lempicki RA (2018). Systematic and integrative analysis of large gene lists using DAVID bioinformatics resources. Nat Protoc.

[47] Kaur R, Sodhi M, Sharma A, Sharma VL, Verma P, Swamiet SK (2018). Selection of suitable reference genes for normalization of quantitative RT-PCR (RT-qPCR) expression data across twelve tissues of riverine buffaloes (Bubalus bubalis). PLoS One.

[48] Bonnet M, Bernard L, Bes S, Leroux C (2013). Selection of reference genes for quantitative real-time PCR normalisation in adipose tissue, muscle, liver and mammary gland from ruminants. Animal..

[49] Jiang Q, Sun B, Liu Q, Cai M, Wu R, Wang F (2019). MTCH2 promotes adipogenesis in intramuscular preadipocytes via an m6A-YTHDF1-dependent mechanism. FASEB J.

[50] Huang J, Dang R, Torigoe D, Li A, Lei C, Sasaki N (2016). Genetic variation in the GDNF promoter affects its expression and modifies the severity of Hirschsprung’s disease (HSCR) in rats carrying Ednrbsl mutations. Gene..

[51] Wei X, Li H, Yang J, Hao D, Dong D, Huang Y (2017). Circular RNA profiling reveals an abundant circLMO7 that regulates myoblasts differentiation and survival by sponging miR-378a-3p. Cell Death Dis.

