# Supplementary figures and images for: Comparative transcriptome analysis reveals that PCK1 is a potential gene affecting IMF deposition in buffalo
Source: BMC Genomics. 2020 Oct 12;21:710. doi: 10.1186/s12864-020-07120-w (PMC7552535; doi:10.1186/s12864-020-07120-w)

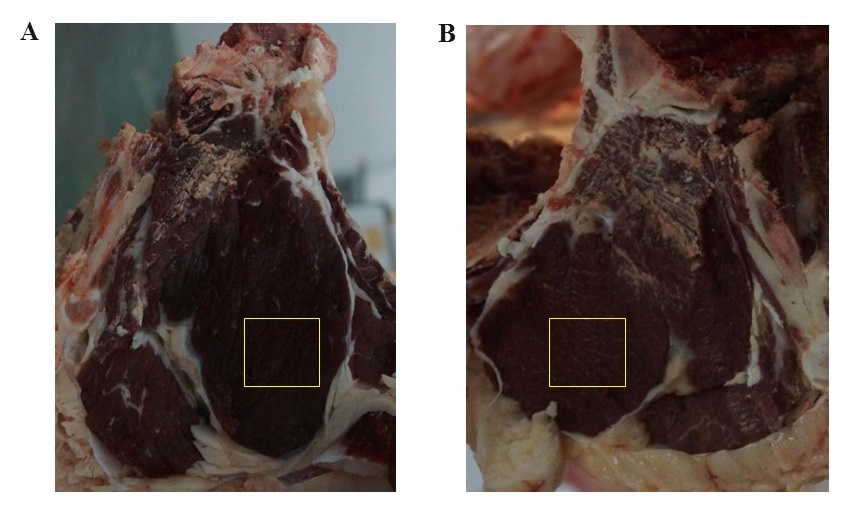

Supplement: Supplementary file 1 — Additional file 1: Fig. S1. Images of the cross-section of the longissimus dorsi muscle in the 12th–13th rib of buffalo (A) and cattle (B). Intramuscular fat (IMF) of the longissimus dorsi muscle is very limited in both buffalo and cattle. IMF in cattle is slightly richer than that in buffaloes. Tissue in the yellow box was sampled for the experiment. [file 12864_2020_7120_MOESM1_ESM.jpg]

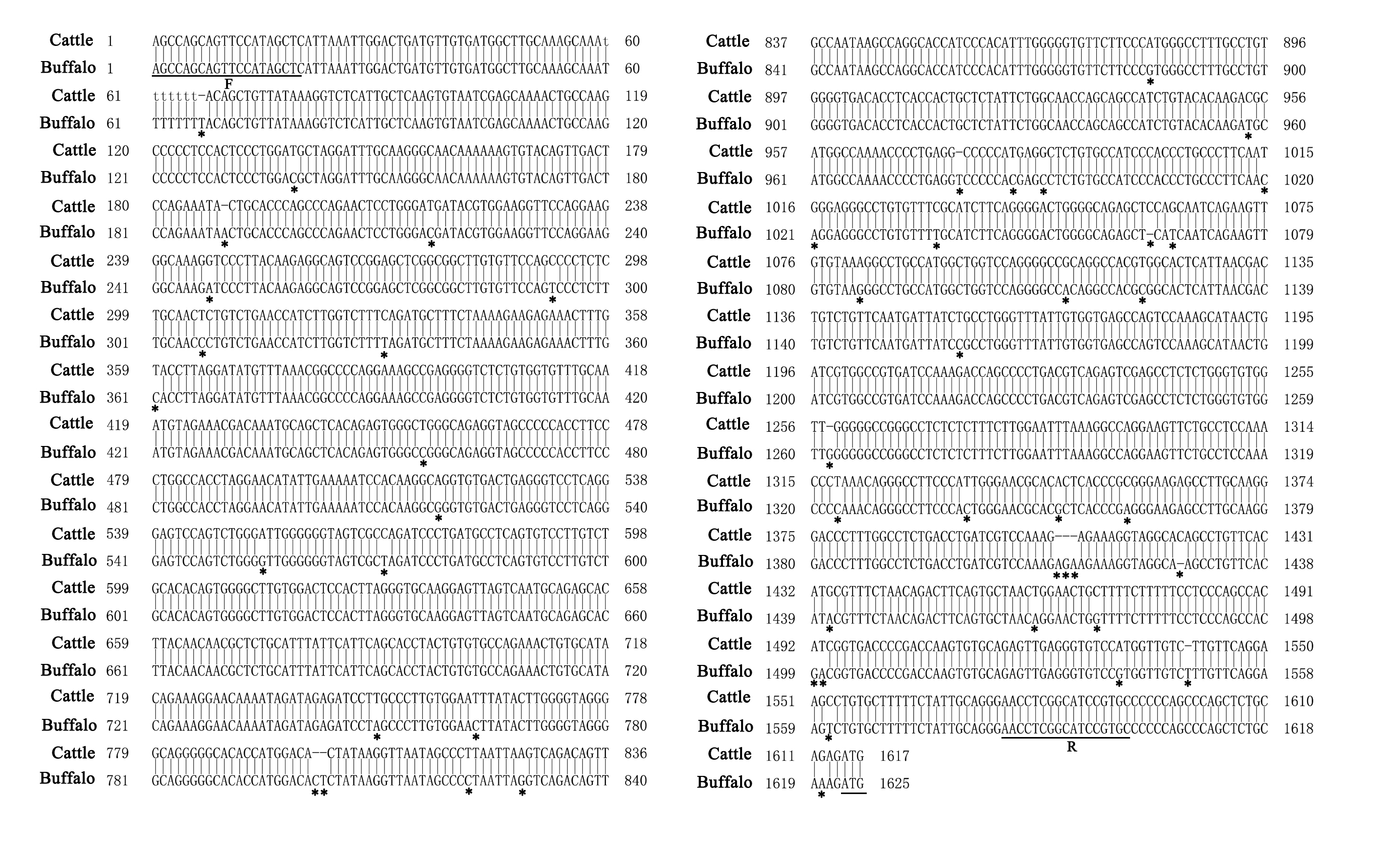

Supplement: Supplementary file 8 — Additional file 8: Fig. S2. Sequences of the upstream region of the buffalo and cattle PCK1 genes. * under sequences indicates base difference between buffalo and cattle. Horizontal lines under sequence indicate the forward primer (F), the reverse primer (R), and the start codon (ATG), respectively. [file 12864_2020_7120_MOESM8_ESM.jpg]
